# Synovial Fluid from Patients with Osteoarthritis Shows Different Inflammatory Features Depending on the Presence of Calcium Pyrophosphate Crystals

**DOI:** 10.3390/ijms25010393

**Published:** 2023-12-27

**Authors:** Francesca Oliviero, Chiara Baggio, Marta Favero, Amelia Carmela Damasco, Carlotta Boscaro, Davide Tietto, Mattia Albiero, Andrea Doria, Roberta Ramonda

**Affiliations:** 1 Rheumatology Unit, Department of Medicine—DIMED, University of Padova, 35128 Padova, Italy; chiara.baggio@unipd.it (C.B.); faveromarta@gmail.com (M.F.); amelia.damasco19@gmail.com (A.C.D.); adoria@unipd.it (A.D.); 2 Medicine Unit 1, Ca’ Foncello Hospital, 31100 Treviso, Italy; 3 Department of Medicine, University of Padova, 35128 Padova, Italy; carlotta.boscaro@unipd.it; 4 Experimental Diabetology Lab, Veneto Institute of Molecular Medicine, 35129 Padova, Italy; mattia.albiero@unipd.it; 5 Fisiored Clinic, 35136 Padova, Italy; ortopediapd@libero.it; 6 Department of Surgery, Oncology and Gastroenterology, University of Padova, 35128 Padova, Italy

**Keywords:** osteoarthritis, synovial fluid, calcium pyrophosphate crystals, inflammation, interleukin-1

## Abstract

The role of calcium pyrophosphate (CPP) crystals in osteoarthritis (OA) is still a matter of debate. With this study we aimed to investigate the inflammatory features of synovial fluid (SF) collected from patients with OA with CPP crystals compared with those without crystals. We also explored the effect of OA SF on monocytes response. SFs were collected from adult patients with OA and subdivided according to the presence of crystals. Local cellular and humoral inflammatory mediators were analysed in the SF samples. The expression levels of IL-1β, IL-18, CASP-1, NLRP3, and GAPDH were measured by RT-PCR in the cells obtained by pelleting the SF samples. For the in vitro study, a monocytic cell line was treated with selected SF samples. SF with CPP crystals showed a significant increase in inflammatory cellular indices and higher levels of IL-1β, IL-8, and caspase-1 transcript with respect to SF without crystals. Higher concentrations of VEGF were also observed in the early stages of the whole OA patients. THP-1 cells stimulated with OA SF released a significant amount of IL-1 β in culture supernatants. This study demonstrated that SF collected from patients with OA shows different inflammatory features depending on the presence of CPP crystals.

## 1. Introduction

Osteoarthritis (OA) is a common arthropathy characterized by persistent pain, stiffness, and reduced joint functions. It is a leading cause of disability worldwide and a great burden for healthcare systems. The most commonly affected joints are the knee, hip, and hand, but most joints can be affected. A recent report on the global burden of OA showed that 595 million people had OA in 2020, corresponding to 7.6% of the global population, and an increase of 132.2% in total cases over the last 30 years [1]. Although the prevalence increases with age, 3.5% of working-aged adults aged 30–60 years experience some form of OA [1]. Among modifiable risk factors, obesity, knee injuries, and muscle weakness seem to play a major role in the development of the disease and represent amenable targets for primary and secondary prevention strategies [2,3]. Treatments for OA are still limited and are addressed to reducing pain and attenuating the excessive mechanical load at the joint. They include both physical and symptomatic pharmacological therapy. Regenerative therapies including mesenchymal stem cells and platelet-rich plasma are used to increase the function of damaged tissues [4] or to enhance the clinical effect of surgical procedures like microfractures [5]. 

Calcium crystals have been proposed to play a crucial role in OA development and progression [6]. Calcium pyrophosphate (CPP) and basic calcium phosphate (BCP) crystals, in particular, have shown a potential inflammatory role in OA pathology [7,8]. These crystals are usually associated with articular cartilage and other joint tissues calcification, as well as meniscus, and lead to different clinical phenotypes including asymptomatic CPP deposition, OA with CPP deposition, acute CPP crystal arthritis with self-limiting synovitis (previously referred to as pseudogout), and chronic inflammatory arthritis [9]. The link between CPP crystals and inflammation has been well established [10]. Through specific signalling pathways, these crystals can stimulate resident cells to produce pro-inflammatory cytokines such as interleukin (IL)-1β and IL-6 that, in turn, promote calcium crystals formation [11] and chondrocytes mineralization. However, in OA their presence is not always associated to articular chondrocalcinosis. Studies on synovial fluid (SF) collected from OA patients without any radiographic sign of chondrocalcinosis have demonstrated that CPP crystals can be found in the early stages of the disease and their presence has been associated with mild local degree of inflammation [12]. Currently, the presence of calcium crystals in OA remains a matter of discussion both for their undefined role in the pathogenesis and in the choice of pharmacological treatment, which is mainly based on pain relief irrespective of the presence of calcium crystals.

With this study we aimed to investigate the inflammatory features of SF collected from OA patients with and without calcium crystals. Consequently, we sought to explore the effect of the synovial OA microenvironment stimulating THP-1 cells with OA SF samples. 

## 2. Results

### 2.1. Synovial Fluid Characteristics

SF characteristics are shown in Table 1. The higher inflammatory degree, in terms of both WBC count and PMN percentage, was found in SF collected from CPP+ patients. Patients without crystals were the youngest among the two groups. 

The grade of radiological OA severity scored according to Kellgren and Lawrence (KL) was available in 35 patients (Table 2). The prevalence of CPP crystals was not different between early (KL = 1 or 2) and late stage of OA (KL = 3 or 4) (Fisher’s exact test, * *p* = 0.32). KL values were not associated with the age of patients (Spearman’s rank correlation test) and with the inflammatory degree of SFs evaluated as WBC and PMN count (Spearman’s rank correlation test).

### 2.2. Cytokines and Chemokines

**Figure 1:**
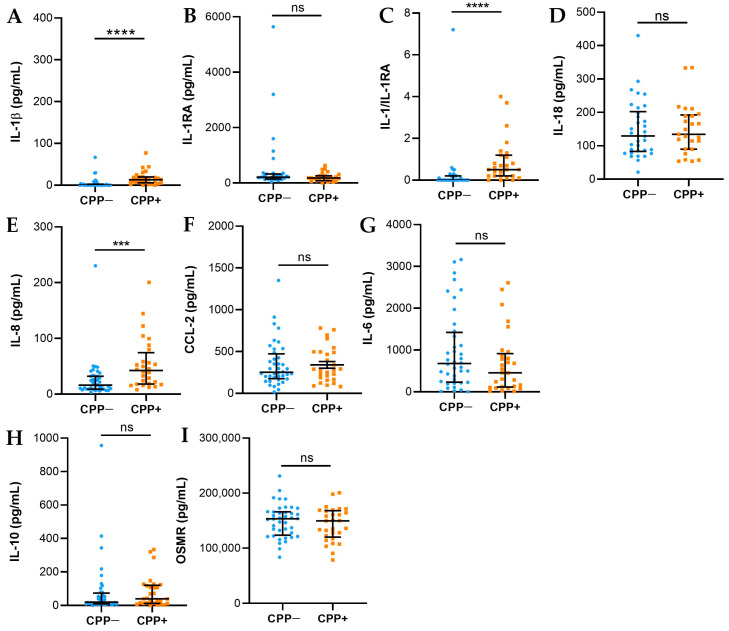
Cytokines and chemokines in SFs from OA CPP+ and OA CPP−. Measurement of IL-1β (**A**), IL-1RA (**B**), IL-1β/IL-1RA (**C**), IL-18 (**D**), IL-8 (**E**), CCL-2 (**F**), IL-6 (**G**), IL-10 (**H**), and OSMR (**I**) levels was performed in 29 SFs from OA CPP+ patients and 39 SFs from OA CPP− patients as described in Materials and Methods. Data are shown as the median (IQR). *p* values calculated according to the Kruskal–Wallis test and Dunn’s post hoc test: *** *p* <0.001, **** *p* <0.0001. Abbreviations are as follows: OA, osteoarthritis; CPP−, SF negative to CPP crystals; CPP+, SF positive to CPP crystals; ns, not significant.

SF cytokines and chemokines are presented in Figure 1. The levels of IL-1β (A) and IL-8 (E) were higher in the SF of CPP+ patients with respect to CPP− patients. Although the levels of IL-1β were higher in CPP+, the levels of IL-18 (D) did not differ between the two groups. Antagonist-free IL-1β (IL-1RA) levels were significantly higher in CPP+ patients than in CPP− patients (C). No significant differences were observed in the levels of CCL-2 (F), IL-6 (G), IL-10 (H), and OSMR (I) between the two groups of patients.

The cytokines/chemokines analysed in SFs from OA patients do not correlate with the KL values and do not differ between groups subdivided according to the KL values analysed (Figure S1).

#### Inflammasome Activation 

The gene expression of NLRP3 and caspase-1 was evaluated by qPCR (Table 2). Although non-significant, the level of caspase-1 transcript was higher in OA CPP+ patients than in OA CPP− patients. NLRP3 expression did not differ between the two groups analysed (Table 3). 

### 2.3. VEGF, BMP-2, MMP-1, and TIMP-1 Levels

**Figure 2:**
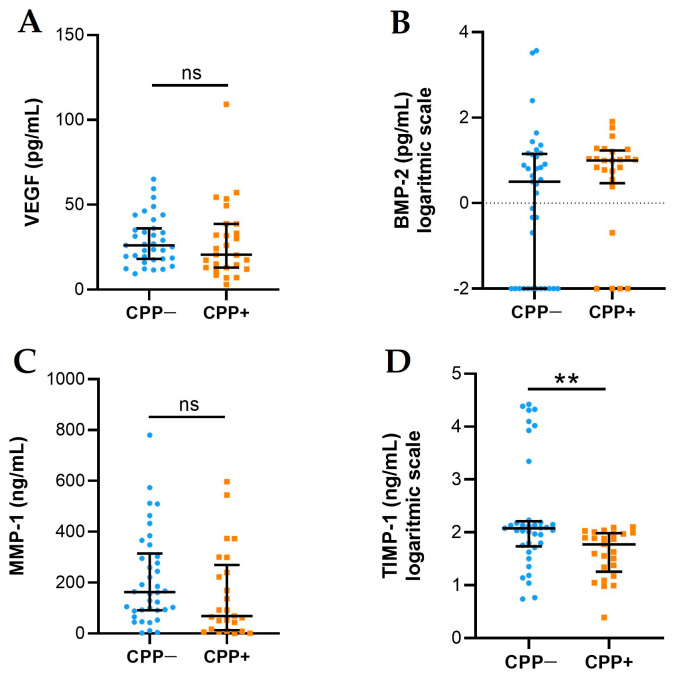
VEGF, BMP-2, MMP-1, and TIMP-1 levels in SFs from OA CPP+ and OA CPP−. Measurement of VEGF (**A**), BMP-2 (**B**), MMP-1 (**C**), and TIMP-1 (**D**) levels was performed in 29 SFs from OA CPP+ patients and 39 SFs from OA CPP− patients as described in Materials and Methods. Data are shown as the median (IQR); BMP-2 and TIMP-1 are shown as logarithmic scale due to their very variable values. *p* values calculated according to the Kruskal–Wallis test and Dunn’s post hoc test: ** *p* < 0.01. Abbreviations are as follows: OA, osteoarthritis; CPP−, SF negative to CPP crystals; CPP+, SF positive to CPP crystals; ns, not significant.

VEGF, BMP-2, MMP-1, and TIMP-1 levels are presented in Figure 2. VEGF (A) and BMP-2 (B) levels did not differ between the two groups. The levels of MMP-1 (C) and TIMP-1 (D) were higher in CPP− patients with respect to CPP+ patients, although significance was reached only for TIMP-1. 

**Figure 3:**
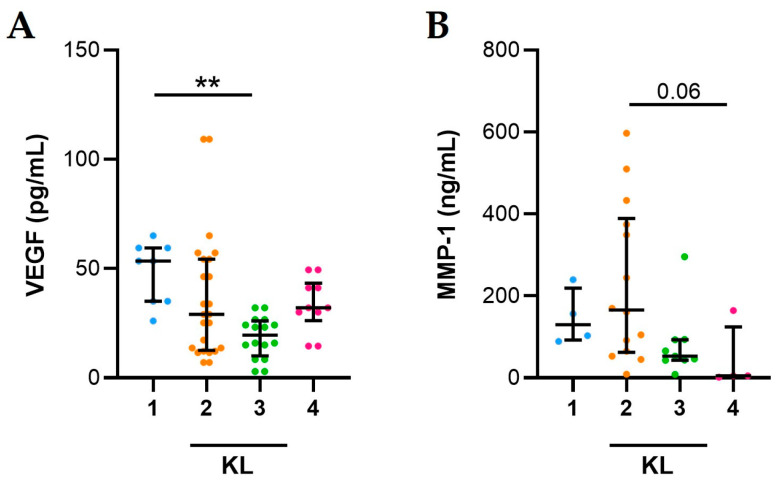
Association between KL values and VEGF and MMP-1 in SFs from OA patients. (**A**) VEGF and (**B**) MMP-1 levels evaluated in 35 SFs from OA patients as described in Materials and Methods. Data are shown as the median (IQR). *p* values calculated according to the Kruskal–Wallis test and Dunn’s post hoc test: ** *p* < 0.01. Abbreviations are as follows: KL, Kellgren and Lawrence classification.

VEGF levels are significantly higher in OA SFs from patients with KL 1 compared to those with KL 3 (Figure 3A). VEGF levels do not correlate with KL score (r = −0.247, *p* = 0.188). Although not significant, MMP-1 levels are higher in OA SFs from patients with KL 2 compared to those with KL 4 (Figure 3B) and MMP-1 negatively correlated with KL score (Spearman’s rank correlation test, r = −0.498, *p* = 0.004).

### 2.4. Association between Inflammation and Synovial Fluid Factors

A positive correlation between local inflammatory cellular indices (including WBC and PMN) and the cytokines IL-1β, IL-8, and IL-10 was found. VEGF was negatively correlated with WBC count and PMN percentage. M was positively correlated with WBC count and PMN percentage and with the levels of OSMR and VEGF, and negatively correlated with IL-1β and IL-8 (Table 4).

### 2.5. Synovial Fluids from Patients with OA Stimulate the Release of IL-1β in Monocytes

**Figure 4:**
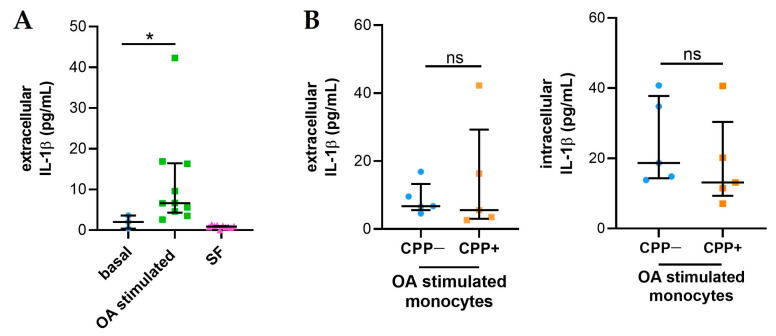
IL-1β released from monocytes treated with SFs from OA patients. THP-1 cells were treated with 20% of SFs (n = 10) for 24h as described in Materials and Methods. (**A**) Levels of IL-1β released from monocytes treated with SFs from OA patients (n = 10). IL-1 β levels were also evaluated in SFs at 20% to stimulate monocytes. Data are shown as the median (IQR). *p* values calculated according to the Kruskal–Wallis test and Dunn’s post hoc test: * *p* < 0.05. (**B**) Intracellular and extracellular IL-1β levels released by monocytes treated with CPP+ OA SFs and with CPP− OA SFs (n = 5). Data are shown as the median (IQR). *p* values calculated according to the Kruskal–Wallis test and Dunn’s post hoc test. Abbreviations are as follows: OA, osteoarthritis; SF, synovial fluid; CPP−, SF negative to CPP crystals; CPP+, SF positive to CPP crystals; ns: not significant.

IL-1β release increased compared to baseline in THP-1 cells treated with 20% of SFs from OA patients (Figure 4A). However, intracellular and extracellular levels of IL-1β released by monocytes treated with CPP+ OA SFs (n = 5) are comparable to those of monocytes treated with CPP− OA SFs (n = 5) (Figure 4B).

## 3. Discussion

The presence of calcium crystals in SF of patients with OA and their role in the disease is still a matter of debate [6,8,9]. According to our previous study, although around 20% OA SF are positive to CPP crystals, it is not clear whether they can influence the progression of the disease or are involved in its development [12]. OA is classically defined as a non-inflammatory arthropathy characterized by painful joints with absent or modest effusion, limited joint function, and the lack of systemic inflammatory indices. OA SF has typical non-inflammatory features with a WBC count lower than 500 cells/mm^3^ [13] and the almost complete absence of PMN cells. However, when CPP crystals are detected, SF display a slight but significant increase in WBC count and PMN percentage while maintaining a non-inflammatory classification (<2000 cells/mm^3^) [14] (Table 1). The capability of CPP crystals to interact with SF cells and induce subclinical inflammation in OA was hypothesized by Martines Sanchis and Pascual, who demonstrated an increased PMN percentage in SF from uninflamed joints of patients with CPP related arthropathies [15]. The presence of a subclinical inflammatory status is also supported by our previous data showing that patients with SF CPP crystals display a positive ultrasound power Doppler signal [12]. In that study, we also found that, despite the higher prevalence in older patients, CPP crystals could also be identified in the early stage of the disease. In the cohort of patients considered in this study, subjects with CPP were older than those without crystals and there was no difference in the radiological score. In the present study, considering the local inflammatory environment, SFs positive to CPP showed higher levels of IL-1β and IL-8 than those without crystals. Remarkably, IL-1 levels showed a positive correlation with PMN percentage and negative with mononuclear cells. This finding supports an important role of PMN in crystal-induced IL-1 protein as previously hypothesized [16]. Furthermore, the expression of caspase-1, responsible for processing the precursor forms of IL-1β into its biologically active form, tended to increase in samples of SF containing CPP crystals.

IL-1β is considered the most active biological substance in crystal-induced inflammation. All pathogenic crystals, including CPP, monosodium urate, and BCP, generate IL-1β through inflammasome dependent and independent mechanisms [17], which, in turn, recruits IL-1 receptor expressing cells such as monocytes and neutrophils. Different chemokines, including IL-8, mediates this process sustaining and amplifying inflammation [18]. Although the levels of these cytokines are not comparable to those found in gout or other crystal-induced arthritis, in OA they might stimulate, at a low degree, cells of the synovial compartment producing detrimental effects over time. 

Considering the recent observation on the activation of vascular endothelial cells in OA synovial fibrosis [19] and their role in subchondral bone angiogenesis [20], we sought to determine VEGF in SF samples, along with a well-known degrading enzyme, MMP-1, associated with vascular remodelling and induced by VEGF itself [21]. Although the levels of the growth factor did not differ among the two populations of patients, we found higher concentrations of VEGF in the early stages of the whole OA patients (KL degree 1–2). This might support the concept that subchondral bone angiogenesis precedes cartilage injury [20]. Furthermore, VEGF was found to correlate with mononuclear cell, an effective source of this growth factor. 

Concerning MMP-1, no difference was observed between SFs with or without CPP crystals. Although we could not verify the activity of the enzyme directly, the higher levels of TIMP-1 in the group of patients without crystals might suggest a decreased activity of MMP-1 itself. 

Finally, in the attempt to reproduce some molecular aspects of OA, THP-1 cells were stimulated with SF samples and IL-1β formation and release was detected. The results showed that OA SF used at a 20% concentration was capable of inducing a significant amount of IL-1 in culture supernatants with no difference between the SF derived from patients with or without crystals. This was probably due to the absence of the physical interaction of crystals with monocytes. Furthermore, that SF could be the active source of this amount was excluded by the determination of IL-1β in those fluids.

This study has some limitations. First, the limited sample size of SFs; although OA is a common condition, patients do not always have effusions, and the aspiration process may depend on patients’ compliance. Furthermore, KL scores were not always available because different images technique were used for clinical evaluation. 

## 4. Materials and Methods

### 4.1. Reagents 

PBS (P4417) and BSA (A4503) were from Sigma-Aldrich (St. Louis, MO, USA) and ABTS (37615) was from Thermo Fisher Scientific (Waltham, MA, USA). ELISA kits for interleukin (IL)-1β (88–7261), IL-6 (88–7066), IL-8 (88–8086), IL-10 (88–7106), CCL-2 (88–7399), OSMR (88–50330), IL-1RA (900-M474), BMP-2 (900-TM255), and TIMP-1 (900-M438) were from Thermo Fisher Scientific (Waltham, MA, USA), VEGF (446504) was from BioLegend (San Diego, CA, USA), IL-18 (DY318-05) was from R&D Systems (Minneapolis, MN, USA), and MMP-1 (ab218184) was from Abcam (Cambridge, UK). The Total RNA purification kit was from Norgen Biotek (Thorold, ON, Canada; 17200). The SensiFAST™ cDNA Synthesis Kit (BIO-65054) and SensiFAST™ SYBR^®^ Lo-ROX Kit (BIO-94050) were purchased from Bioline (London, UK).

### 4.2. Study Design and Patient’s Characteristics

Synovial fluids (SFs) were collected by arthrocentesis from swollen knees of untreated adult patients with OA (n = 69) who attended the outpatients’ clinic of the Rheumatology Unit at Padua University Hospital. The diagnosis of OA was made by expert rheumatologists based on signs and symptoms suggestive of OA according to EULAR recommendations [22] and was confirmed by the SF characteristics (white blood cell count lower than 2000/mm^3^). We divided the SF samples by the presence or absence of CPP crystals detected at the compensated polarized light microscopy. We included in the study 39 patients without SF calcium pyrophosphate crystals (CPP−) and 29 patients with SF calcium pyrophosphate crystals (CPP+). Patients’ demographic and SF characteristics are shown in Table 1. OA grade was scored by Kellgren and Lawrence (grade from 0 to 4) in the x-rays available (n = 35) [23].

Discarded samples were stored and studied under protocols approved by the local Institutional Review Board and after have obtained the subjects informed consent (approval #39872).

### 4.3. Synovial Fluid Examination

**Figure 5:**
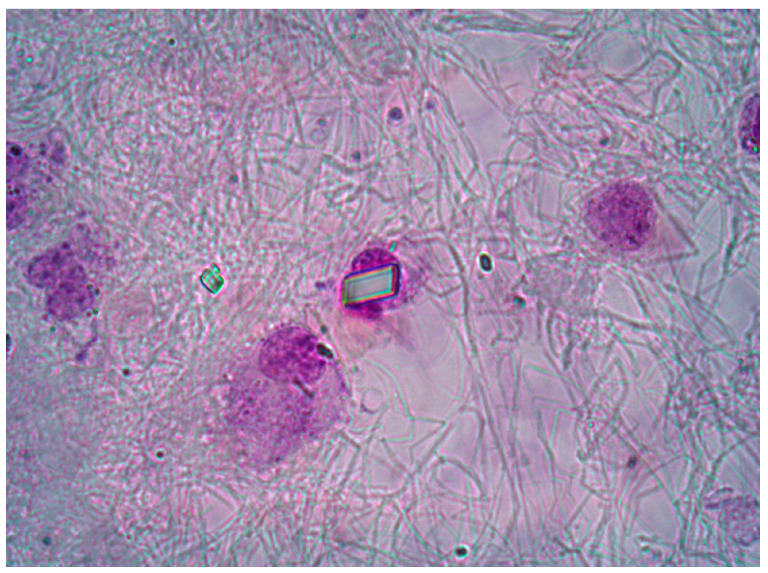
Intracellular CPP crystal observed in OA patient using a pre-stained slide. An extracellular rhomboid crystal is also visible. Ordinary light microscopy. 1000× magnification.

SFs samples were collected in anticoagulant and plain tubes and analysed by ordinary and polarized light microscopy. A total of 39 SF CPP− and 29 SF CPP+ samples were tested in the study. Total white blood cell count (WBC) count was performed using a Bürker counting chamber. Differential leucocyte count, providing the percentage of polymorphonuclear (PMN), monocytes (M), and lymphocytes (L) in the SF, was performed using pre-stained slides for cell morphology (Testsimplets^®^). Crystal examination was performed under compensated polarized light microscopy. Monosodium urate (MSU) and calcium pyrophosphate (CPP) crystals were identified by their shape, degree, and sign of birefringence [24]. A typical rectangular CPP crystal is shown in Figure 5. After analysis, SF samples were centrifuged at 800 rpm for 20 min to collect cells (lower partitioning layer) and supernatant and stored at −20 °C until further examinations. 

### 4.4. Cytokine, Chemokine, Growth Factor, Metalloproteases, BMP-2, and TIMP-1 Measurement

The following mediators were measured in SF after proper PBS dilutions using commercially available enzyme-linked immunosorbent assay (ELISA) kits: interleukin (IL)-1β (dilution 1:1, sensitivity: 2 pg/mL), IL-6 (dilution 1:20, sensitivity: 2 pg/mL), IL-8 (dilution 1:10, sensitivity 2 pg/mL), IL-10 (dilution 1:1, sensitivity 2 pg/mL), CCL-2 (dilution 1:10, sensitivity 7 pg/mL), OSMR (dilution 1:10, sensitivity 312.5 pg/mL), IL-1RA (dilution 1:1, sensitivity 23.4 pg/mL), BMP-2 (dilution 1:1, sensitivity 15.62 pg/mL), TIMP-1 (dilution 1:1, sensitivity 15.62 pg/mL) (Thermo Fisher Scientific, Waltham, MA, USA), VEGF (dilution 1:1, sensitivity: 7.8 pg/mL; BioLegend, San Diego, CA, USA), IL-18 (dilution 1:1, sensitivity 11.71 pg/mL; R&D Systems, Minneapolis, MN, USA), and MMP-1 (dilution 1:100, sensitivity 156.25 pg/mL; Abcam, Cambridge, UK). 

### 4.5. RNA Extraction and Real-Time qPCR

Selected SF samples (CPP+ n=3, CPP− n=6) were centrifuged at 1500 rpm for 10 min to collect cell pellets and finally stored at −20 °C. A Total RNA purification kit (Norgen Biotek, Thorold, ON, Canada) was used to isolate RNA according to the manufacturer’s recommendations. RNA was quantified with a NanoDrop™ 2000 Spectrophotometer (Thermo Fisher Scientific, Waltham, MA, USA). A SensiFAST™ cDNA Synthesis Kit (Bioline, London, UK) was used to synthesize cDNA. The expression levels of IL-1β, IL-18, CASP-1, NLRP3, and GAPDH were measured by real-time quantitative PCR (qPCR) using a SensiFAST™ SYBR^®^ Lo-ROX Kit (Bioline, London, UK) via Quant Studio 5 real-time PCR system (Thermo Fisher Scientific, Waltham, MA, USA). Genes were normalized to GAPDH and analysed using the 2−∆Ct method. The primer sequences are reported in Table 5. 

### 4.6. Cell Culture and Treatment with Synovial Fluids

The monocytic human cell line THP-1 were seeded in a flat 96-well plate at a density of 50,000 cells/well in RPMI 1640 containing 10% FBS and supplemented with 1% glutamine (Sigma-Aldrich, St. Louis, MO, USA) and 1% penicillin-streptomycin (Sigma-Aldrich, St. Louis, MO, USA) (complete medium) and incubated overnight at 37 °C, 5% CO_2_. The following day, THP-1 cells were stimulated with 20% of SFs from OA patients CPP+ (n = 5) and CPP− (n = 5) in RPMI supplemented with 1% glutamine and 1% penicillin-streptomycin and cultured for 24 h. The presence of SF also allowed cell priming as previously demonstrated [25]. Subsequently the culture supernatants were collected, centrifuged at 1300 rpm for 5 min, and frozen for subsequent determinations of extracellular IL-1β by ELISA kit. Intracellular IL-1β was determined by ELISA in lysates obtained after three freeze–thaw cycles and resuspended in PBS.

### 4.7. Statistical Analysis

The Shapiro-Wilk test was used to analyse the distribution of continuous variables, and variables with a non-normal distribution were presented as medians with the corresponding interquartile range (IQR). As no variable was normally distributed among the categories, only non-parametric tests were used. The differences between the two groups of patients (CPP− vs. CPP+) were tested using the Mann–Whitney U test. The Kruskal–Wallis test followed by Dunn’s post hoc tests were used for multiple comparisons. Categorical variables were compared using the Chi-square test. Spearman correlation analysis was used to determine correlations. Statistical analysis was performed using GraphPad Prism version 8 (GraphPad Software Inc., La Jolla, CA, USA). A *p* value < 0.05 was considered significant.

## 5. Conclusions

This study demonstrated that SF collected from patients with OA shows different inflammatory features depending on the presence of CPP crystals. These patients have higher local cellular inflammatory indices including WBC and PMN, and increased levels of IL-1β and IL-8. The in vitro study confirmed the subclinical inflammatory microenvironment in OA. At present, we do not have enough data to establish if CPP crystals are an epiphenomenon or an inducer of OA, and the scientific literature is very scarce in this regard. However, the inflammatory status associated with the presence of crystals might define patients at major risk to progress towards a more severe disease and allow a more tailored treatment to be developed for patient phenotypes.

## Figures and Tables

**Table 1:** Characteristics of the patients included in the study and their SF.

OA	CPP−	CPP+	*p*
Patients, n	39	29	
Age, years (IQR)	66 (52–76)	74 (68–79)	0.0235
Sex, n	F, 27; M, 12	F, 19; M, 10	ns
WBC, n/mm^3^ (IQR)	100 (100–200)	200 (100–300)	0.0028
PMN, % (IQR)	0 (0)	2 (0–9.5)	<0.0001
M, % (IQR)	98 (98)	96 (90–98)	<0.0001

Data are expressed as the median and interquartile range (IQR). Categorical variables were compared using the χ2 test; *p* values calculated according Mann–Whitney test. Abbreviations are as follows: CPP−, SF negative to CPP crystals; CPP+, SF positive to CPP crystals; OA, osteoarthritis; WBC, white blood cell; PMN, polymorphonuclear cells; M, monocytes; IQR, interquartile range; ns, not significant.

**Table 2:** Kellgren and Lawrence classification of OA patients.

Score, n	CPP− (n = 20)	CPP+ (n = 15)
1–2	13 (65%)	7 (46.6%)
3–4	7 (35%)	8 (53.4%)

Abbreviations are as follows: CPP−, SF negative to CPP crystals; CPP+, SF positive to CPP crystals.

**Table 3:** Caspase-1 and NLRP3 gene expression in SF from OA with (CPP+) and without (CPP−).

	CPP−	CPP+	*p*
Caspase-1, 2-∆Ct (IQR)	0.1366 (0.02409–0.2733)	0.2292 (0.1568–0.5294)	ns
NLRP3, 2-∆Ct (IQR)	0.1240 (0.1136–0.1414)	0.1130 (0.01196–0.1167)	ns

Gene expression was performed in selected SFs (CPP− n = 6, CPP+ n = 3) as described in Materials and Methods. Data are expressed as the median and interquartile range (IQR). *p* values calculated according to the Kruskal–Wallis test and Dunn’s post hoc test. Abbreviations are as follows: IQR, interquartile range; CPP−, SF negative to CPP crystals; CPP+, SF positive to CPP crystals; ns, not significant.

**Table 4:** Correlations between SF cytokines, chemokines, growth factor, MMP-1, and TIMP-1 levels and total and differential white blood cell count.

	WBC	PMN	M
IL-1β	ns	*p* = 0.001 (r = 0.419)	*p* = 0.049 (r = −0.253)
IL-8	*p* = 0.001 (r = 0.387)	*p* < 0.0001 (r = 0.556)	*p* < 0.0001 (r = −0.504)
IL-10	*p* = 0.014 (r = 0.299)	ns	ns
OSMR	ns	ns	*p* = 0.015 (r = 0.309)
VEGF	*p* = 0.039 (r = −0.267)	*p* = 0.007 (r = −0.362)	*p* = 0.031 (r = 0.291)

Spearman’s rank correlation test. Abbreviations are as follows: WBC, white blood cell; PMN, polymorphonuclear cells; M, monocytes; ns, not significant.

**Table 5:** Primer sequences used for gene expression study.

Gene	Forward Primer	Reverse Primer
IL-1β IL-18 NLRP3 CASPASE-1 GAPDH	CAGCCAATCTTCATTGCTCA TGTCGCAGGAATAAAGATGGCT TGAAGAAAGATTACCGTAAGAAGTACAGA GCTGAGGTTGACATCACAGGCA TGCACCACCAACTGCTTAGC	TCGGAGATTCGTAGGTGGAT CCTTGGTCAATGAAGAGAACTTGGT GCGTTTGTTGAGGCTCACACT TGCTGTCAGAGGTCTTGTGCTC GGCATGGACTGTGGTCATGAG

## Data Availability

Data are contained within the article.

## Supplementary Materials

The following supporting information can be downloaded at: https://www.mdpi.com/article/10.3390/ijms25010393/s1.
