# Supplementary material for: Synovial Fluid from Patients with Osteoarthritis Shows Different Inflammatory Features Depending on the Presence of Calcium Pyrophosphate Crystals
Source: Int J Mol Sci. 2023 Dec 27;25(1):393. doi: 10.3390/ijms25010393 (PMC10778695; doi:10.3390/ijms25010393)
Supplement: Supplementary file 1 [file ijms-25-00393-s001.zip › ijms-2750394-supplementary.pdf]

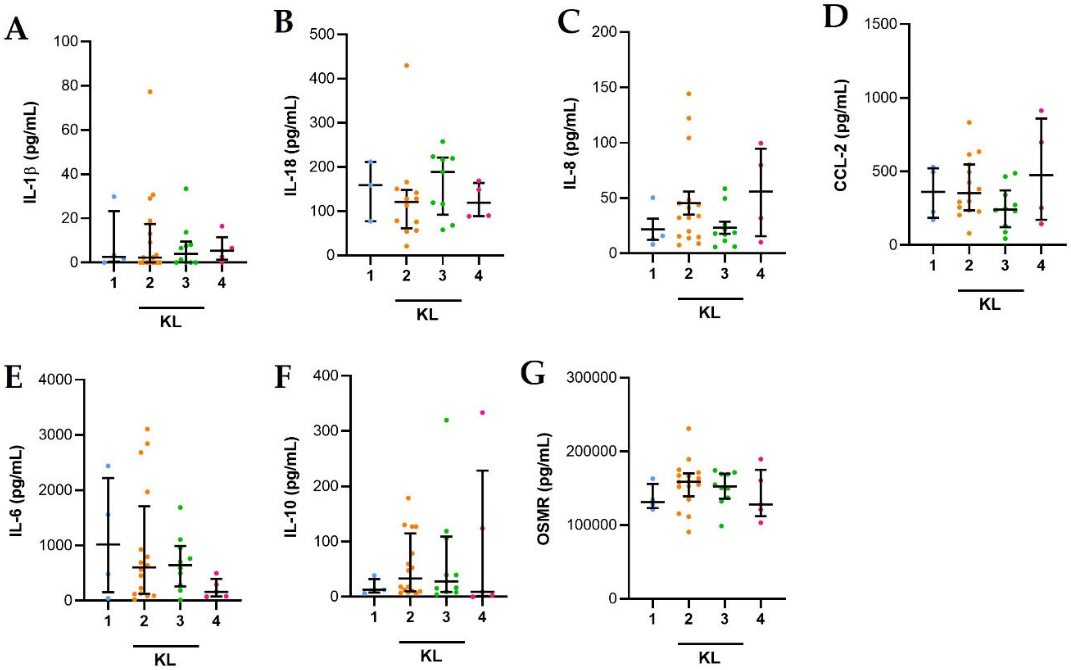

Supplementary Figure S1. Association between KL values and cytokine-chemokine in SFs from OA patients. (**A**) IL 1β, (**B**) IL-18, (**C**) IL-8, (**D**) CCL-2, (**E**) IL-6, (**F**) IL-10 and (**G**) OSMR levels was performed in 35 SFs from OA patients as described in Materials and Methods. Data are shown as the median (IQR). p calculated according to the Kruskal-Wallis test, Dunn's post hoc test. Abbreviations are as follows: KL, Kellgren and Lawrence classification.
